# Fluorescence confocal microscopy for margin assessment in prostatectomy: IP8‐FLUORESCE study protocol

**DOI:** 10.1111/bju.16588

**Published:** 2024-11-16

**Authors:** Nikhil Mayor, Alexander Light, Anna Silvanto, Emma Cullen, Peng Yun Ng, Almostafa Badreldin, Bijan Khoubehi, Giles Hellawell, Francesca Fiorentino, Martin J. Connor, Taimur T. Shah, Hashim U. Ahmed, Mathias Winkler

**Affiliations:** ^1^ Imperial Prostate, Division of Surgery, Department of Surgery and Cancer, Faculty of Medicine Imperial College London London UK; ^2^ Department of Urology Imperial College Healthcare National Health Service (NHS) Trust London UK; ^3^ Department of Pathology University College London Hospitals NHS Foundation Trust London UK; ^4^ Department of Urology Chelsea and Westminster NHS Foundation Trust London UK; ^5^ Nightingale‐Saunders Clinical Trials & Epidemiology Unit, King's Clinical Trials Unit & Division of Methodology King's College London London UK

**Keywords:** prostate cancer, radical prostatectomy, surgical margins, novel technology, fluorescence confocal microscopy, confocal laser microscopy

## Abstract

**Background:**

Radical prostatectomy (RP) represents the cornerstone of surgical treatment for prostate cancer. Assessing surgical margin status intraoperatively with current techniques remains challenging due to high costs in the context of an already stretched pathology workforce. Fluorescence confocal microscopy (FCM) is a promising technique to detect margins in prostate cancer surgery not bound by such limitations.

**Study Design:**

The Imperial Prostate 8 – Fluorescence Confocal Microscopy for Rapid Evaluation of Surgical Cancer Excision (IP8‐FLUORESCE) study is a multicentre, prospective, *ex vivo*, ‘blinded’, comparative cohort study. It aims to assess the accuracy of digital FCM for detection of prostate cancer at surgical margins compared to traditional histopathology.

**Endpoints:**

The primary endpoint is the accuracy of digital FCM for detection of prostate cancer at surgical margins on a per‐patient level, reported with sensitivity, specificity, positive and negative predictive values.

**Patients and Methods:**

A total of 153 patients with localised prostate cancer undergoing robot‐assisted RP across three UK National Health Service tertiary referral centres will be recruited. Following RP, prostate specimens will undergo immediate immersion in Acridine Orange solution, scanning ‘en face’ with FCM using the Histolog® Scanner, and subsequent formalin fixation and paraffin embedding. Two independent, ‘blinded’ uro‐pathologists will report both the FCM images and the histopathology slides. Recruitment commenced on 17 August 2023.

**Abbreviations**
FCM: fluorescence confocal microscopy
H&E: haematoxylin and eosin
ICHNT: Imperial College Healthcare NHS Trust
IP8‐FLUORESCE: Imperial Prostate 8 – FLUOrescence confocal microscopy for Rapid Evaluation of Surgical Cancer Excision (study)
NeuroSAFE: neurovascular structure‐adjacent frozen‐section examination
NVB: neurovascular bundle
PSM: positive surgical margin
RP: radical prostatectomy

## Background

Approximately 5000 radical prostatectomies (RPs) for localised prostate cancer are performed each year in the UK, with that number set to increase [1]. In well‐selected patients, it is an effective oncological procedure, but functional outcomes are variable with some studies reporting high rates of urinary incontinence and erectile dysfunction [2, 3]. Preserving the neurovascular bundle (NVB) in nerve‐sparing RP can spare erectile function in those with adequate preoperative potency but the improvement in functional outcome may come at the expense of cancer control, with potential for higher rates of positive surgical margins (PSMs). A significant proportion of men with a PSM will experience biochemical recurrence, which in turn may require additional adjuvant treatment [4]. Whilst prospective evidence that PSMs heighten mortality risk is lacking, analysis of large retrospective datasets has shown an association of PSMs with disease‐specific and overall mortality in those with adverse tumour characteristics [5].

The only well‐studied method for intraoperative assessment of PSMs is the neurovascular structure‐adjacent frozen‐section examination (NeuroSAFE) technique using frozen sections [6]. By evaluating margin status intraoperatively, the NeuroSAFE technique offers surgeons the opportunity to perform a re‐resection of the NVB if a PSM is identified. It has been proven to reduce PSMs, although the long‐term oncological benefits remain unknown [6]. Critically, the technique can allow more men to undergo nerve‐sparing RP and potentially benefit from the associated improved functional outcomes [7]. The NeuroSAFE PROOF trial (ClinicalTrials.gov identifier: NCT03317990) aims to definitively prove the functional improvements associated with intraoperative margin assessment [8]. Despite these promises, NeuroSAFE has never been widely adopted. The technique is expensive and requires specialist machinery (such as a cryostat) that can only be operated by trained laboratory staff. Coupled with the nationwide shortage of histopathologists and the requirement to prolong RP operating times by ~1 h, the NeuroSAFE technique has not become the standard of care and is offered by few UK surgeons [6, 9, 10]. There is therefore a requirement for a cheaper, faster, and similarly accurate method of real‐time PSM assessment during prostate cancer surgery that does not rely on expert technicians: fluorescence confocal microscopy (FCM) is the foremost candidate of all real‐time tissue imaging technologies.

### What is FCM?

Confocal laser microscopy uses pinhole apertures in the microscope detector to reject out‐of‐focus light, reducing blur and producing high‐resolution imaging in thick tissue samples [11]. FCM is the addition of a fluorescent dye, which further enhances the cell‐to‐stroma contrast. In recent years, ultrafast digital scanning confocal microscopes have been developed allowing rapid visualisation of microscopic structures. Novel FCM scanners have an increased field‐of‐view, which allows whole tissue specimens to be scanned without the need for specimen dissection or fixation. The technique requires minimal tissue preparation (typically immersing samples in soluble Acridine Orange for 10 s then rinsing with saline) and can produce high‐resolution images in <1 min. Scanners are compact and portable and can therefore be kept in the operating theatre or an office. Notably, the samples can be re‐examined with conventional techniques after FCM as the fluorescent dye does not affect the specimen integrity and tissue fixation is not required. Images can be directly uploaded to electronic patient records for review by pathologists or trained urologists [12].

### Existing Evidence

**Fig. 1:**
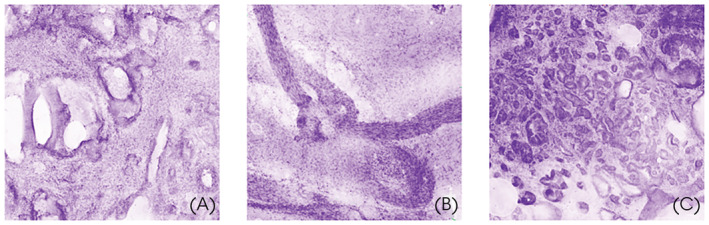
High‐resolution digital FCM images of (**A**) benign glandular tissue; (**B**) NVB; (**C**) cancerous tissue.

Fluorescence confocal microscopy appears to be highly accurate when compared to traditional histological analysis across multiple organ systems [13]. In the setting of skin cancer, the technique is well‐established, allowing for rapid identification of margins with high sensitivity and specificity in Mohs samples [14]. The potential application in intraoperative real‐time analysis of surgical margins in RP has been analysed in four small studies including just over 100 patients in total (Table 1) [12, 15, 16, 19]. These studies serve as proof‐of‐concept and highlight the excellent agreement between FCM and histopathology. It is also evident that the technique is significantly faster than NeuroSAFE (8 vs 50 min) [12]. Initial reports have highlighted the excellent discrimination between benign glandular tissue, the NVB, and neoplastic regions (Fig. 1). Further, the ‘en face’ scanning method described by Almeida‐Magana et al. [16] is a welcome advancement, meaning no tissue sectioning is required and the capsule of the specimen remains intact for conventional histopathological reporting. Nevertheless, none of the studies were ‘blinded’, nor were they powered to draw key conclusions on accuracy of FCM in RP.

**Table 1:** Summary of evidence: FCM in RP.

Author	Rocco et al. [15]	Baas et al. [12]	Almeida‐Magana et al. [16]	Musi et al. [19]
Design	Proof‐of‐concept Prospective, single‐centre case series	Prospective, single‐centre, comparative, feasibility study	Proof‐of‐concept Prospective, single‐centre case series	Non‐randomised exploratory study (secondary analysis of phase III single‐centre RCT)
Blinding	Unblinded – pathologist aware of outcome from conventional histopathology when reporting FCM images	Unblinded	Unblinded	Pathologists ‘blinded’ from each other ‘Blinded’ reporting of FCM, histopathology, and NeuroSAFE not documented
FCM technology	VivaScope 2500M‐G4 FCM (Mavig GmbH, Munich, Germany)	Histolog® Scanner (SamanTree Medical SA, Lausanne, Switzerland)	Histolog Scanner	VivaScope 2500M‐G4 FCM
FCM technique	Mohs shaving of apex and posterolateral margins	Mohs shaving of posterolateral margins	Intact scanning of posterolateral margins	Mohs shaving of suspicious
Comparator	Conventional histopathology	NeuroSAFE and conventional histopathology	Conventional histopathology	NeuroSAFE and conventional histopathology
Number of patients	24	50 patients	31 patients	45 patients
		96 margins	60 margins	54 margins
Procedure time	15–40 min	Median (IQR) 8 (5–20) min	<10 min	15–18 min
Secondary resection	Focal/wedge in cases of positive FCM	Complete resection of NVB in cases of positive NeuroSAFE	None performed	Partial/full resection of NVB in cases of positive NeuroSAFE
Outcome	4 PSMs identified, converted to NSM with secondary resection 1 PSM outside of intraoperatively assessed region	NeuroSAFE: 14 (15%) PSMs Sensitivity: 93% Specificity: 99% CLM: 15 (16%) PSMs Sensitivity: 86% Specificity: 96% Agreement between NeuroSAFE and CLM: κ = 0.80	CLM: 7 (12%) PSMs Sensitivity: 87.5% Specificity: 98.1% Agreement between CLM and histopathology: κ = 0.86	Moderate (κ = 0.74) to almost perfect (κ = 0.90) agreement between pathologists dependent on scoring method Sensitivity 70.5–81.0%, specificity 65.0–91.8% for most experienced pathologist dependent on scoring method Moderate (κ = 0.62) to strong (κ = 0.86) agreement between FCM and NeuroSAFE for most experienced pathologist dependent on scoring method

CLM, confocal laser microscopy (synonymous with FCM); IQR, interquartile range; κ, kappa coefficient; NSM, negative surgical margin; RCT, randomised controlled trial.

The dearth of well‐designed studies in this field means that most men undergoing RP are not benefitting from the potential advantages of intraoperative margin assessment. The Imperial Prostate 8 – FLUOrescence confocal microscopy for Rapid Evaluation of Surgical Cancer Excision (IP8‐FLUORESCE) study is the first robustly‐powered, multicentre, ‘blinded’ study to evaluate the accuracy of FCM for detection of PSMs in prostate cancer surgery. Here, we describe in detail the study protocol.

## Study Design

**Fig. 2:**
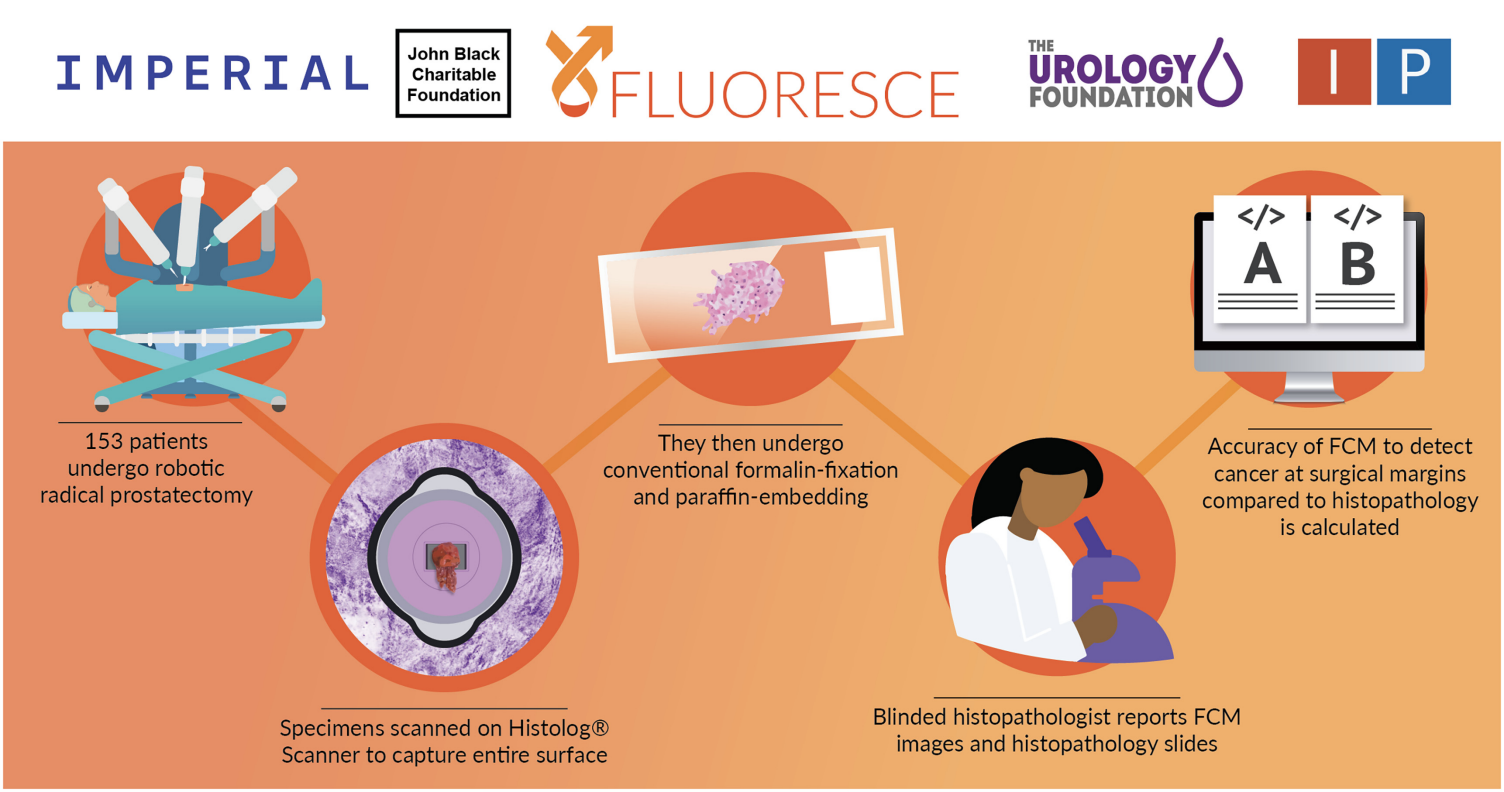
Study flow diagram.

The IP8‐FLUORESCE study is a multicentre, prospective, *ex vivo*, ‘blinded’, comparative cohort study in which a minimum of 153 patients will be recruited across three UK NHS tertiary referral centres offering high‐volume robot‐assisted prostatectomy services. The study is sponsored by Imperial College Healthcare NHS Trust (ICHNT). The lead centre for the study is ICHNT, with patients also being recruited from University College London Hospitals NHS Foundation Trust (UCLH) and Guy's and St Thomas’ NHS Foundation Trust (GSTT). The main objective of the study is to determine the accuracy of digital FCM in detecting and ruling out PSMs in prostate specimens on a patient level, in patients undergoing RP for prostate cancer, compared to the histopathological ‘gold standard’ [17]. Fig. 2 shows the study overview.

Ethical approval for the study was granted independently by the tissue biobanks associated with each centre: Imperial College Healthcare Tissue Bank (Project Reference: R23015); UCL Human Tissue Biobank (NC36.24); King's Health Partners Cancer Biobank (24‐PUB‐NM‐01). The study is generously funded by The John Black Charitable Foundation and The Urology Foundation (Innovation and Research Award 2023). The study will be conducted according to the ethical principles stated in the Declaration of Helsinki and with the principles of The International Council for Harmonisation of Technical Requirements for Pharmaceuticals for Human Use (ICH) Guideline for Good Clinical Practice (GCP). The study was prospectively registered on the International Standard Randomised Controlled Trial Number Register (ISRCTN21536411).

### Outcome Measures

The primary outcome is accuracy (reported with sensitivity, specificity, positive and negative predictive values) of digital FCM for detection of prostate cancer at surgical margins, with traditional haematoxylin and eosin (H&E) histopathology as the reference standard, on a per‐patient level.

Secondary outcomes include:

- Sensitivity, specificity, positive and negative predictive value of digital FCM for detection of cancer at surgical margins (any PSM and significant PSMs), with traditional H&E histopathology as the reference standard, on a per‐image/margin level.
- Cancer detection by digital FCM (any PSM and significant PSMs), determined using area under the receiver operating characteristic curve, with traditional H&E histopathology as the reference standard.
- Agreement of digital FCM with the pathology report for cancer length (mm) at the surgical margin on a margin and patient level.
- Agreement of digital FCM with the pathology report for cancer grade at margin on a margin and patient level.
- Cohen's kappa coefficient for agreement between readers (two individual consultant histopathologists; consultant histopathologist vs trainee histopathologist; consultant histopathologist vs urologist)

### Eligibility Criteria

#### Inclusion Criteria

- Any patient aged >18 years with a prostate undergoing robot‐assisted RP at any of the participating centres during the study period.
- Both nerve‐sparing and non‐nerve‐sparing cases will be included, with no restriction on surgeon technique (e.g., Retzius‐sparing) or type of robotic operating system used.

#### Exclusion Criteria

- Patients unwilling or unable to consent to *ex vivo* use of tissue for research purposes.
- Any patient undergoing salvage prostatectomy (i.e., after pelvic radiotherapy for prostate cancer, or any focal ablative therapy such as high‐intensity focused ultrasound, cryoablation, or irreversible electroporation).

### Methods

#### Procedure

Patients with localised prostate cancer diagnosed based upon suspicious MRI with associated confirmatory prostate biopsy whose case has been discussed in the locoregional multidisciplinary meeting will undergo robot‐assisted RP. Both nerve‐sparing (bilateral or unilateral) and non‐nerve‐sparing procedures will be performed depending on individual surgeon assessment of each case. RP will be performed by seven consultant urological surgeons across the three major tertiary referral study centres with a median (interquartile range) experience of 16 (9–17) years in performing independent RP.

All participants scheduled for RP undergo routine history taking, examination, diagnostic evaluation, and staging to ensure suitability for the procedure. All included patients will undergo standard‐of‐care preoperative evaluation including renal and liver function testing, electrocardiography, and anaesthetic review. Due to the *ex vivo* design of the study, no screening visits are required Fig. S1. Further, patients will not have any contact, undergo any tests, or require any follow‐up out of line with standard‐of‐care management other than the digital FCM scan.

#### The FCM Technique

The Histolog^®^ Scanner (SamanTree Medical, Lausanne, Switzerland) is a Conformité Européene‐*in vitro* diagnostic (CE‐IVD)‐certified scanning device with a wide‐field‐of‐view confocal laser scanning microscope, designed for scanning large biological specimens in a clinical setting. Tissue fluorescence is excited by a laser at the wavelength of 488 nm and fluorescence emission is collected at a wavelength >500 nm. Additional post‐processing is not required though an inbuilt system automatically converts the digital images from monochromatic to purple in order to increase readability and align more closely with traditional H&E‐stained images.

Prostate specimens will be extracted from the abdominopelvic cavity and immediately immersed in an Acridine Orange solution for 10 s, then rinsed in 0.9% saline and transferred to the Histolog Scanner. The specimens will be scanned ‘en face’, without the need for any *ex vivo* tissue resection. After positioning on the Histolog Scanner, specimens weighed down with a small bag of sand to increase tissue contact with the scanning surface. A live preview image allows the operator to make a technical judgement of the completeness of the image, the presence of excess tissue, and the absence of air bubbles. If necessary, the specimen will be repositioned, and a high‐resolution digital image will be acquired within 50 s. The procedure will be performed for six margins (base, apex, left and right posterolateral, anterior, posterior) in total for each specimen in order to scan the entire tissue surface.

The specimen will then be formalin‐fixed and paraffin‐embedded for standard‐of‐care histological assessment. Clinical management of the patients will follow current standard‐of‐care pathways. No intraoperative decisions (e.g., re‐resection) will be made based on assessment of margins with FCM in this study.

### Outcome Reporting

Once recruitment is complete, histopathology slides for each patient will be pseudoanonymised and stored securely. Two independent, expert uro‐pathologists will report both the FCM images, and the histopathology slides unaware of the outcome of the opposing histological/imaging technique (i.e., when reporting an FCM image they will be unaware of the histopathology result, and vice versa). The histopathologists will undertake a standardised training module devised by SamanTree Medical, as well as a review of 30 previous cases undertaken in a pilot study at one of the study centres. For each case, the pathologist will receive a case report form detailing the patients’ presenting PSA, MRI result, biopsy outcome, and a description of the macroscopic specimen. A blank pathology case report form is shown in Fig. S2. Each histopathologist will provide the overall margin status on a per‐patient level, as well as on an image/margin level, for length of cancer at the margin and highest grade.

#### Definitions

*Definition 1*. Any PSM.

Any length of prostate cancer of any grade present at the inked margin on conventional histopathology.

*Definition 2*. Significant PSM.

The presence ≥3 mm length of any Gleason pattern OR primary Gleason pattern ≥4 of any length OR the presence of multifocal positivity of any grade or length. The decision to use this definition was based upon a review of the literature pertaining to clinically significant PSMs [18]. There was consensus amongst the histopathologists involved in the study that this definition encompasses the commonly accepted definitions of significant PSMs in prostatectomy.

#### Sample Size Determination

After review of the literature for key parameters, an a priori sample size calculation was carried out. The sample size was calculated to estimate robustly the sensitivity of FCM to detect cancer. This is in line with the practical applications of this diagnostic test in the setting of RP, where the aim would be to introduce FCM intraoperatively to trigger a change in management (e.g., wider resection margin or excision of the NVB) if a PSM was detected. Based on outcomes with the Histolog Scanner in the few studies published, a desirable sensitivity of 0.85 was assumed in the sample size estimation [12, 15, 16].

#### Prevalence of PSMs

Prevalence of PSMs varies widely in the literature, influenced heavily by the definition of a PSM (e.g., some authors only report PSMs if greater >3 mm), the risk profile of the local population, PSA screening penetrance, average tumour volumes in prostate specimens, and surgeon experience amongst numerous other factors. To estimate the prevalence of PSMs in the study population, we performed a retrospective review of 768 consecutive RPs including very high risk and locally advanced cases at the lead study centre. The overall PSM rate was 41%; 47% for high‐risk cancers, 37% for intermediate‐risk cancers and 34% for low‐risk cancers. As the true prevalence of PSMs for the study cohort was not known before commencing recruitment, an interim assessment of observed PSM rate after each consecutive 50 patients had been recruited was planned a priori in order to adjust the sample size accordingly. The assessment was based on the clinical histopathology reports for each patient, which includes PSM status and was carried out by a pre‐specified independent ‘blinded’ researcher.

For the sample size calculation, before commencing recruitment, the following assumptions were made:

- Sensitivity: 0.85.
- Prevalence of PSM: 30%.
- Precision: ±14 points.
- Confidence level: 95%.
- Expected dropout rate for scans with inadequate image quality: 10%.

The estimate for sensitivity was based upon the largest published comparative series from Baas et al. [12] who report a sensitivity of 86% based upon 96 posterolateral RP specimens analysed with FCM compared, unblinded, to definitive histopathology. The estimated sample size for the precision of the sensitivity was the presence of 27 PSMs, inflated by 10% for expected drop out. Adjusting for the assumed prevalence of 30% of PSMs, a sample size of 100 patients was deemed sufficient.

#### Interim Prevalence Review 1

After 50 patients had been recruited, the estimated prevalence of PSMs was 43%. Given that the prevalence at the interim event rate review was higher than estimated initially, the Trial Management Group agreed to increase the precision margin of the sensitivity estimate to ±10 points to increase the robustness of the study conclusions. This led to an adjusted sample size estimate of 130 patients. A further event rate review was planned after 100 patients.

#### Interim Prevalence Review 2

After 100 patients, the prevalence was 41%. Adjusting for the slight reduction in prevalence and accounting for expected dropout, an updated recruitment target was calculated at 140 patients (to maintain the precision margin of the sensitivity estimate to ±10 points).

#### Detection of Significant PSMs

In order to detect significant PSMs at an assumed prevalence of 18% (based on the Interim Prevalence Review 2) and a precision level of ±14 points, a final target of 153 patients was set.

#### Methods of Data Collection

Baseline demographic data (e.g., age, ethnicity, comorbidities), patient risk factors (e.g., DRE findings, family history of prostate cancer, serum PSA level at presentation), and tumour characteristics (e.g., MRI findings, biopsy result) will be prospectively entered into a secure database by a member of the research team at each site. Data and all appropriate documentation will be stored for 10 years after the completion of the study.

After having undergone standard‐of‐care reporting, histopathology slides will be collected from the laboratory, manually pseudoanonymised, and stored securely until re‐reported by the independent pathologist on a pre‐designed case report form (Fig. S2). All specimens will be handled by members of the research team with appropriate clinical training in line with the Human Tissue Act 2004.

#### Statistical Analysis Plan

Full details of planned statistical summaries and analyses will be outlined in a pre‐defined, signed, and dated statistical analysis plan before any analyses are performed. Any deviations from the analysis plan will be fully documented with reasons for doing so.

#### Timeline

Recruitment commenced on 17 August 2023. At the time of protocol publication, more than half of patients have been recruited to the study. Analysis of FCM images will only occur when recruitment has been completed, at which point the database will be locked. Results are expected to be available for publication in Spring 2025.

## Discussion

Surgeons are performing more RPs than ever before. It is imperative, therefore, that incremental gains are made wherever possible to improve cancer control and quality of life for patients undergoing RP. Surgeons are obligated to offer all patients nerve‐sparing RP where possible, and intraoperative assessment of margins should mean that more patients are eligible. Frozen section with the NeuroSAFE technique has proven accurate but not feasible due to major cost implications and resource burden in an already stretched pathology workforce. FCM with the Histolog Scanner is an ultrafast digital imaging technique that could be a game‐changer for intraoperative margin assessment. The IP8‐FLUORESCE study is the first study to robustly assess the accuracy of this novel technology in a ‘blinded’, multicentre setting.

The prospective, multicentre design is a notable strength, offering robustness and generalisability to our findings. Further, it is the first study to ‘blind’ the histopathologist to the outcome of the histopathology results when reporting FCM images. Here, we also publish our a priori power calculation. By incorporating interim event rate reviews, we have been able to ensure the study remains adequately powered at all stages.

The unexpectedly high PSM rate observed in our study population, attributed to the high admixture of high‐risk and locally advanced patients from the local unscreened population, serves as an unexpected yet beneficial aspect of our study. Whilst high PSM rates could raise concerns, it bolsters the power of our study by allowing the use of a narrower precision margin in the final sample size estimation. PSM rates are closely correlated with tumour volumes and are a median of 2.5 mL in this cohort with a PSA screening penetrance of <8%.

Whilst this study is well‐placed to assess the accuracy of FCM in detecting PSMs, the fact remains that PSMs are a surrogate marker for long‐term oncological outcomes with limited prospective evidence. Moreover, the clinical significance of a PSM continues to be debated, given the variability in tumour biology and the complex multifactorial pathway leading to prostate cancer progression. Notably, no two margins are equivalent, with factors such as capsular breach adding further complexity to their interpretation.

The ‘en face’ technique employed in our study, whilst efficient in its ability to scan the entire tissue surface without tissue sectioning, raises questions regarding its clinical significance compared to traditional cross‐sectional techniques. Further investigation into the implications of PSMs assessed ‘en face’ is warranted to better understand the impact on oncological outcomes and on guiding clinical decision‐making.

Finally, we acknowledge that our study primarily aims to assess the accuracy of FCM for PSM detection and does not provide direct insight into functional or oncological outcomes. Future studies incorporating comprehensive assessments of functional and oncological endpoints with re‐resection in the case of PSMs, as well as economic analysis, are warranted to fully elucidate the clinical utility of FCM in prostate cancer surgery.

## Disclosure of Interests

Nikhil Mayor is supported by funding from the UK National Institute for Health Research (NIHR). He has received funding from The Urology Foundation and The John Black Charitable Foundation for work pertaining to this study. Alexander Light is supported by funding from the UK NIHR and has received funding from The Urology Foundation for work outside the scope of this study. Taimur T. Shah receives infrastructure support from the NIHR Imperial Biomedical Research Centre (BRC) and Imperial College ECMC. Hashim U. Ahmed is supported by core funding from the UK NIHR Imperial BRC and the Imperial NIHR/Cancer Research UK (CRUK) Experimental Cancer Medicine Centre. He currently receives funding from the Wellcome Trust, Medical Research Council (UK), CRUK, Prostate Cancer UK, NIHR (UK), and Imperial Health Charity, for trials in prostate cancer. He is a proctor for high‐intensity focused ultrasound (Sonablate Inc.), cryotherapy (Boston Scientific) and Rezum (Boston Scientific) and is paid for training other surgeons in these procedures. He is a paid scientific advisory board member for Francis Medical; has given lectures for Boston Scientific, Ipsen, and Janssen; and has received funding to attend scientific conferences from Janssen. He has been on a medical advisory board for Janssen in the last 3 years but not currently.

## Supporting information


**Fig. S1.** Imperial College Healthcare Tissue Bank patient information sheet.

**Fig. S2.** Pathology case report form.
