# Supplementary material for: Fluorescence confocal microscopy for margin assessment in prostatectomy: IP8‐FLUORESCE study protocol
Source: BJU Int. 2024 Nov 16;135(3):502–9. doi: 10.1111/bju.16588 (PMC11842882; doi:10.1111/bju.16588)
Supplement: Supplementary file 1 — Fig. S1. Imperial College Healthcare Tissue Bank patient information sheet. [file BJU-135-502-s001.pdf]

Imperial College Healthcare NHS
NHS Trust

Tissue Bank

Information for patients, relatives and carers version 8 20/07/2022

Introduction

This leaflet provides information about the Imperial College Healthcare Tissue & Biobank (ICHTB) run by Imperial College London and Imperial College Healthcare NHS Trust. We hope it will help you to understand:

- the aims and purposes of ICHTB
- what research is likely to be performed
- what your participation (if you agree to it) will involve

Please read the following information carefully and feel free to ask us if anything is unclear or if you would like more information: telephone 020 3311 7173 or email us via tissuebank@imperial.ac.uk

What is ICHTB?

ICHTB is a project that will help the Trust and its partner, Imperial College London, carry out high quality healthcare-related research to better understand diseases and improve future treatments.

ICHTB aims to provide researchers with access to a very large resource of human tissue samples that are vital in helping us to:

- better understand your health conditions
- reach our goal of developing new medicines and other treatments
- improve ways of detecting diseases earlier

By taking part, you will be helping researchers investigate and understand why some individuals have or develop a particular disease, and to understand the biology of the disease so we can develop better treatments.

In addition, healthcare professionals may need to study tissue or fluids as part of their training. It is hoped that this knowledge will help other patients in the future.

We also want to give you the option to take part in further research studies related to your condition or that are relevant to you. To do this we need to be able to contact you to provide further details of these studies so you can decide if you want to take part. NHS clinical staff will contact you to explain the study and ask for your

permission to share your contact details with the researcher running it. You have the right to say no.

Do I have to join ICHTB?

No. It is completely up to you whether or not you wish to join. If you decide not to it will not affect the standard of care that you receive in any way. If you do decide to join, you will be free to withdraw at any time and without having to give a reason.

Allowing us to keep your biological samples for research

When you attend hospital your care team will often need to perform tests and procedures to diagnose and treat your condition. These procedures may involve the removal of fluids, pieces of tissue or sometimes a whole organ (known as biological samples). These biological samples are sent to a laboratory to be tested by doctors or scientists. This can help them diagnose your condition.

Not all of the biological samples may be needed for diagnosis. If you consent to join ICHTB, any leftover biological samples after diagnosis may be kept for research (under normal circumstances these would be thrown away).

If you are having a biopsy procedure, two or three extra samples may be taken for research which may mean the procedure would take a few extra minutes. If you have already had your operation you can still help us by consenting - we can then ask the pathologist for a small amount of tissue already used for your diagnosis.

After treatment, your doctor may invite you back for further visits and this may involve taking other biological samples. If, at a later stage, you have blood or tissues taken as part of your future care, we may wish to access these samples too. This will allow researchers to make potentially helpful comparisons with your original samples. If you do not wish to provide further samples, you do not have to.

Allowing us to take additional blood samples

As part of your regular hospital treatment, a doctor may need to take blood samples from you. If you consent to ICHTB, when you provide these blood samples, we may take up to an additional 45 millilitres (ml) - three tablespoons - of blood to be stored for research. You will not need to attend any extra appointments - we will only take this extra blood when you are required to provide blood samples as part of your regular clinical care. If you do not wish to provide further samples, you do not have to.

How will I benefit from joining ICHTB?

You may not benefit personally from any research carried out using your samples and data. However, the results of the research may benefit patients with your type of illness in the future. You will not receive a financial reward now or in the future for providing samples. The medical team involved in your treatment and care will receive no payment because of your donation.

Your sample will not be sold for profit to researchers, but the use of your samples may lead to the development of new drugs, treatments or tests by both academic and commercial organisations

Will the samples be tested for (genetic) inherited disorders?

We want to know how genes influence disease. Genes are made out of DNA. We may, now or in the future, isolate, analyse and store a sample of your DNA from your donated biological samples. Using current advanced laboratory techniques or those developed in the future, we may determine your genetic make-up. This could include determining the sequence of all or part of your DNA code.

ICHTB will not disclose information about your genetic make-up in any way that could harm you or your family. Your personal identifiable information will never be shared without your permission.

Who will use the stored samples?

Your sample may be used by researchers based in academic institutions, the NHS or commercial companies worldwide. All research will be thoroughly reviewed by a tissue management committee before being allowed access to your samples and data. This committee will ensure that the research is scientifically valid, in the interests of human health and has appropriate security measures to protect your information. All of your information will be anonymised which means it will not contain details that could identify you. Your identity will never be shared without your permission.

What will happen to the results of the study?

Research studies usually take several years to complete. The results from these studies will be used to improve treatment and care of patients in the future. Results will be published as appropriate in scientific papers and magazines and regular updates of research in progress, research results and other relevant information will be published on the Imperial College London website at www.imperial.ac.uk/tissuebank.
You will not be identified in any publication or through any information on the website.

What if you find something new about my health?

Results for individual patients from particular research studies will not normally be given to you or your doctor. In very rare situations, some research projects could identify changes to your diagnosis or treatment or may indicate an inherited disease that could affect you or your family members. Your hospital doctor will be notified of this information.

When and how will I be contacted about research studies that I may wish to join?

Approved researchers will be able to search your anonymised health data, data obtained from your biological samples, and other data approved for use in research to find people who may be suitable to take part in research studies. For example, a search might be carried out to find people who are over the age of 40 and have diabetes. If your data matches the requirements for a research study that may be

beneficial to you, we will allow an NHS clinician at Imperial College Healthcare NHS Trust to contact you and provide you with more information. If you are interested in finding out more, the NHS clinician will pass your contact details on to the researcher who will get in touch with you to discuss it further.

You decide if you want to take part. It is your choice and you do not have to take part in any studies if you do not wish to. If you decide not to take part it will not affect the standard of care that you receive in any way.

What will happen to my data if I join ICHTB?

Your information is protected under the Data Protection Act 2018. We have a legal duty to keep your information confidential, secure and hold the information only as long as necessary. Identifying data such as name, NHS number and contact details, will only be available to Tissue Bank Staff members who hold an NHS contract via the consent form you have signed, which we have to keep on record to demonstrate you have agreed to participate.

The Trust collects and stores information about you in order to provide safe and effective healthcare. Your health records are held on paper and on secure computer systems. The Trust allows clinical Information from health records to be used to conduct ethically approved research and this health information may be linked to tissue samples you provide, this data includes test results, surgery and chemotherapy events and diagnosis information. If you agree to take part in the study, the information about your health and care may be provided to researchers running other research studies in this organisation and in other organisations within the UK or overseas.

Identifying data such as name, NHS number and contact information will not be made available to researchers and researchers will not be able to identify you, as the donor or be able to find your personal details, this allows research to occur without impacting patients privacy.
The Trust will use your contact details to tell you about research studies that may be beneficial to you based on your health information. You do not have to take part in any studies if you do not wish to.

You have a right to a copy of the information that we hold about you. To request this information please write to the Subject access requests team via the details listed at the end of this leaflet.

I would like to join ICHTB – what should I do next?

If you have been approached by one of trained consenters in the clinic, just let them know that you wish to consent and they will help you enrol via our secure IT system. If you are discussing consent with an Imperial College Healthcare NHS Trust clinician at the hospital they will show you how to join ICHTB.

I no longer want to be a member of ICHTB – what do I need to do?

You can leave ICHTB at any time without giving a reason and without your medical care or legal rights being affected. We will remove your details from the database and destroy any biological samples that we still have in storage from you for

research and teaching. If you withdraw sometime after joining, some of your samples may have already been used in research.

If you want to leave ICHTB please contact the team directly on 020 3311 7173 or via email at tissuebank@imperial.ac.uk

Who has approved/reviewed the collection of human tissue samples as part of ICHTB?

A Research Ethics Committee based in Wales (Wales REC3) has approved and will regularly review this project.

This project follows the UK Policy Framework for Health and Social Care Research which sets out principles of good practice in the management and conduct of health and social care research in the UK.

Contact details for the data protection officer

Post: Data protection officer, Information Governance, ICT Directorate, Charing Cross Hospital, London, W6 8RF
Email: Imperial.dpo@nhs.net
Telephone: 020 3311 7344

Enquiries relating to Subject Access Requests (DPA) should be sent to - imperial.accesstohealthrecords@nhs.net

Imperial College Healthcare NHS Trust is a registered data controller under the Information Commissioner's Office. Further information can be found at:
Information Commissioners Office, Wycliffe House, Water Lane, Wilmslow, Cheshire, SK9 5AF
Website: www.ico.org.uk/concerns
Phone: 0303 123 1113

If you are not satisfied with our response or believe we are processing your personal data not in accordance with the law you can complain to the Information Commissioner's Office

Contact details for Imperial College Healthcare's patient advice and liaison service (PALS)

If you have any questions or comments about your care, please contact PALS on **020 3313 0088** (Charing Cross, Hammersmith and Queen Charlotte's & Chelsea hospitals), or **020 3312 7777** (St Mary's and Western Eye hospitals). You can also email PALS at pals@imperial.nhs.uk The PALS team will listen to your concerns, suggestions or queries and is often able to help solve problems on your behalf.

Alternative formats

This leaflet can be provided on request in large print, as a sound recording, in Braille, or in alternative languages. Please contact the communications team on **020 3312 5592**.
