# Supplementary material for: Fluorescence confocal microscopy for margin assessment in prostatectomy: IP8‐FLUORESCE study protocol
Source: BJU Int. 2024 Nov 16;135(3):502–9. doi: 10.1111/bju.16588 (PMC11842882; doi:10.1111/bju.16588)
Supplement: Supplementary file 2 — Fig. S2. Pathology case report form. [file BJU-135-502-s002.pdf]

FLUORESCE

Patient ID: e.g *XX123*

PSA:

MRI findings:

Biopsy result:

Macroscopic Description:

	**Histopathology Margin Status**		
	0=negative<br>1=positive<br>2=indeterminate	*Length (mm)*	*Grade (highest; 3, 4, 5)*
Apex			
Lateral (left)			
Lateral (right)			
Anterior			
Posterior			
Base			
**Overall**			

	**Histolog® Fluorescence Confocal Microscopic Images Margin Status**		
	0=negative<br>*1=positive*<br>*2=indeterminate*	*Length (mm)*	*Grade (highest; 3, 4, 5)*
Apex			
Lateral (left)			
Lateral (right)			
Anterior			
Posterior			
Base			
**Overall**			
